# A Practical Work on the Diseases of the Eye, and Their Treatment, Medically, Topically, and by Operation

**Published:** 1841-01-01

**Authors:** 


### A Practical Work on the Diseases of the Eye, and their Treatment, Medically, Topically, and by Operation. By *Frederick Tyrrell*, &c. &c.

[Continued.]

In our last number, we introduced this excellent practical work to our readers, and brought under their notice its author's opinions and practice in reference to the ophthalmiæ. We shall now take up the volume again, and glean here and there the practical hints which abound in it. For, as we observed on a former occasion, *practical* merit is its characteristic.

We first turn to some morbid conditions of the conjunctiva, not coming within the class of the positive ophthalmiæ.

*Polypi of the Conjunctiva*, may be elevated by a pair of forceps, and separated from the conjunctiva, by means of the scissors, or knife; after which, the nitrate of silver, in substance, should be applied to the surface of the wound, and weak astringent lotions frequently used.

*Pterygium.*—Membranous, or fleshy, it appears to result from continued chronic inflammation—is frequent in warm climates, rare in this or colder ones.

As long as the pterygium does not extend to the surface of the cornea, local stimuli and astringents are best for it. If the surface of the cornea has become implicated, nothing but an operation is of service.

"A slender bladed knife should be passed between the pterygium and the sclerotic coat, having the cutting edge towards the cornea; the pterygium should then be separated from the sclerotic, as far as the margin of this transparent structure; in which situation the edge of the knife should be made to divide the morbid texture, so as to leave the portion in connexion with the cornea untouched; the flap thus formed over the sclerotic, should be elevated by a pair of forceps, and the separation of the pterygium from the globe completed, as far as its base or outer attachment, when it should be cut off, either with the knife or scissors, close to the sound membrane; a little care is afterwards requisite to prevent an inflammatory process, and a reproduction of the disease; and I have usually found the simple astringents, as the solution of alum and of the acetate of lead, sufficient for this purpose, keeping the patient perfectly quiet, until the healing process has been completed." 189.

Soon after the operation, the portion of pterygium left over the cornea, gradually disappears. Should the operator have incautiously excised it, an opaque deposit subsequently is developed.

*Fræna.*—Uniting opposed surfaces of the conjunctiva, common as they are after the introduction of lime into the eye, would seem to be given up by Mr. Tyrrell as a bad job.

"For such disease, I believe there is little that can be done, either by medical or surgical aid;—after the occurrence of the injury, which I have described as giving rise to this affection, I have repeatedly tried, by the greatest care and attention, to prevent the formation of fræna; and although, in some instances, I have succeeded in preventing the immediate inosculation of the granulations, yet I have never been able to prevent the formation of a frænum; it has appeared to me, that a contraction of the cicatrix takes place after the healing process is completed, similar to that we so frequently see in the extensive cicatrices, formed after burn of the integuments. I have, with the utmost pains and patience, effected the removal of such bands, and watched the after process of cure; but am now convinced, after repeated trials, that such operations are worse than useless, as they cannot be accomplished without severe suffering, and do not eventually, at all benefit the patient. In two cases, after excising the band or frænum, I kept a very thin and smooth piece of silver constantly between the eyelid and globe, so as effectually to prevent inosculation of granulations, as the surfaces healed. When perfectly healed, much good appeared to have been effected; but, in less than six months, contraction had taken place of the new-formed matter, and fræna were developed as bad as, or worse than those which I had removed." 193.

*Ecchymoses of the Conjunctiva.*—When the extravasation is extensive, or the action of the absorbents very tardy, Mr. Tyrrell has recourse to a light poultice, made by mixing some of the black-briony root, scraped finely with a little crumb of bread. This is placed in a muslin bag, over the palpebræ, for several hours together; and, usually, it has an excellent effect in promoting the action of the absorbent vessels.

*Escharotic Substances in the Eye.*—Immediately after the introduction of lime or mortar into the eye, great relief will be obtained by the free ablution of the organ with weak vinegar and water, in the proportion of about a tea-spoonful of the former, to half a pint of the latter. It should be used warm, and if the means are at hand, by injecting a portion of it into the eye, its beneficial effects will be increased. Otherwise, some of the solution may be dropped into the eye. The good effects of the application result from its chemical action on the lime, which destroys its caustic property and renders it soluble, so that it no longer acts as an irritant. After injury from acids, some alkaline preparation, in solution, will be most serviceable, as a solution of the carbonate of soda or potash ; or, in case such are not to be readily got at, common soap and water will prove a good substitute. These applications will be most serviceable if injected into the eye, or dropped freely upon the conjunctiva.

When heated metal has occasioned the mischief, all that can be done immediately, is to remove the particles that may adhere to the conjunctival surface, or lodge beneath the eyelids.

Subsequently, the treatment must, in all cases, be similar, and such as is calculated to moderate excessive inflammation.

### Affections of the Cornea.

1. *Corneitis.*—Mr. Tyrrell's description of this is very good. But it is not requisite to transcribe it. We may observe, however, that functional disturbance materially affects it, and more particularly deficient cutaneous action. Mr. T. has also sometimes found, in young and delicate females, that error in the periodical uterine secretion, has had an important effect over the corneitis. It is also modified by the condition of general power.

In the treatment, he recommends the influence of mercury, but gently administered, and closely watched. He would begin usually, in children, with one grain of mercury with chalk, and generally combine with it two or three grains of the compound powder of ~~antimony.~~ ×The frequency of the ×Scam dose must depend on the power of the patient, and it may be necessary to intermit it. Besides the exhibition of the mercurial, in most instances, it is necessary to give some tonic remedy; and the selection of this, must depend upon the condition of the principal secretions, or of the circulating fluid. Where the secretions of the digestive organs are regular, and the patient merely wants power, the preparations of bark, as quinine, or the solution of yellow bark, will be found of service; but if, at the same time, with the want of power, the patient is unusually pallid, and the extremities cold, some preparation of steel, will effect more good than the bark. In cases occurring with a marked scrofulous diathesis, he has prescribed small doses of iodine, with hydriodate of potass in some light bitter infusion.

From amongst several cases, we select one. It is, perhaps, related a little diffusely, but as an illustration, it is not without its value.

"I lately saw a nobleman, who had been the subject of corneitis in both eyes, for above five months. It occurred in the latter part of the winter, and whilst he considered his general health to be good; but he had, for a week or two previously, experienced unpleasant rheumatic pains, about his shoulders and neck. The means, resorted to, at first, to relieve the ocular disease, were altogether of a depletory kind; and, under such treatment, the disease advanced. Other advice was then called for, and he took colchicum and mercury, and used further means of depletion locally. He still got worse. Another opinion was then obtained, and mercury was prescribed in large and frequent doses, with a continuance of leeching and cupping locally, with a spare diet. The mercury produced diarrhœa, and great general depression and debility, so much so, that it could not be continued. The hydriodate of potash, with iodine, was also given, but it produced distress of stomach, and could not be gone on with. A more generous diet was then allowed; leeches were occasionally applied to the eyelids; and counter-irritation was promoted by daily friction on the temples. His general health now improved, and the ocular affection became, in a degree, mitigated; and, in this state, I was requested to see him. I found considerable intolerance of light; numerous conjunctival and sclerotic vessels, filled with red blood; both corneæ nebulous, but not sufficiently so, to prevent my ascertaining that the irides were grey or blue. I could not detect vessels, filled with red blood, on the cornea. The patient could discern the outline of a person, but could not distinguish features. His general aspect was pallid, with a look of depression. The hands were rather cold, and the pulse quick but feeble, and the skin felt somewhat harsh and dry. I recommended a continuance of generous diet, and of such stimulus as was found agreeable and beneficial; that he should take one grain of mercury with chalk, and two of compound powder of antimony, night and morning, (there was no evidence of mercurial action at this time,) with some cusparia and ammonia, twice a day; that he should be immersed in a warm bath every other day, for ten minutes at a time, at a temperature not exceeding 98°; that he should be allowed exercise in the open air daily, when the weather was dry, and the wind not from the east or the north. I further consented to the continuance of slight counter-irritation, on the temples, by friction; and the application of a leech or two, to the lower eyelid, in case of a recurrence of pain; and a drop of a solution of belladonna, (which had been used for some days previously,) was also to be applied as before. After eight days I saw the patient again, and found very considerable improvement. The intolerance of light was greatly diminished, and on my approaching him, he said he could distinguish my features, so as to be able to recognise me, in future, with facility. The red conjunctival vessels in the left eye had nearly disappeared; and the cornea, to rather more than half the upper part, had regained its transparency. Nebulæ still existed at the lower part, so that he could not distinguish objects below the eye. The right eye still exhibited some degree of conjunctivitis and sclerotitis, and the entire cornea remained hazy, but not so densely so, as on my previous visit. He had not pursued the plan of treatment recommended to its full extent, inasmuch as he had not taken more than two or three doses of the cusparia and ammonia; and, after two or three days from my first visit, he had omitted one dose of the mercurial in the day, which he had been desired to do, if he had any symptoms of disturbance of stomach or bowels; and this he had warning of; but in diet, exercise, and use of the bath, he had been most regular: and he applied two leeches, on two occasions, to the lower lid of the right eye. A continuation of the same plan was urged, and I saw him again, after the interval of a week. His progress, during the interval, had been most satisfactory; a further improvement having taken place, both as regarded the local disease and the general health. He had, however, taken a few doses of the hydriodate of potash, by the advice of his surgeon, and this had created a slight degree of gastric disturbance. It was, therefore, agreed that it should be omitted, whilst, in every other respect, the treatment was to be continued as before. After a week's interval, again I found still further progress to recovery, the left cornea being nearly clear, and the right presenting only slight nebula at its lower and outer part; but the vessels of the conjunctiva and sclerotic still exhibited, in a trifling degree, unnatural distention by red blood. The progress had been so steady and satisfactory, that we considered our patient might safely quit London, which he did a few days afterwards; I saw him just before he left town, and found little more than slight nebula of the right cornea remaining." 225.

*Of Inflammation of the Cornea with Vesication.*—We notice Mr. Tyrrell's account of this affection—a partial separation of the conjunctiva of the cornea, by effusion of serous fluid.

The symptoms, says Mr. Tyrrell, occur in paroxysms, and there is an extremely irritable condition of the eye; great intolerance of light, severe darting pains, a sensation, as if a sharp and hard foreign particle, were lodged beneath the palpebra; increased heat and lachrymation, creating a sense of scalding. These attacks are gradual in their approach, being preceded by slight uneasiness; but they subside suddenly, leaving only a dull aching pain, which is augmented towards evening.—There is not any useful vision, even between the paroxysms.

Spasmodic action of the orbicularis muscle, from exposure to light during the paroxysm, and a discharge of the superabundant fluids; evidence of increased action in the conjunctiva and sclerotic, many of their vessels being filled with red blood; only few of the latter, but very many of the former, being perceptible. A partial nebulous condition of the cornea exists, in the centre of which, a small vesicle distended with fluid, may be perceived, if the eye be examined during the paroxysm; otherwise, a thin portion of loose membrane, which is partly separated, much as the cuticle is, after the puncture of a cutaneous vesicle.

Mr. T. has only seen this disease in adults, out of health, and, in most instances, when there has been a tendency to rheumatic affections. Local remedies he has seen no benefit from, and it is only from constitutional treatment that he has seen any. A case will illustrate *that.*

"In the first well-marked case of the kind which came under my care, I tried numerous local applications, but without producing any good; and I also pursued several plans of general treatment, for a long time, but could not effect any decided improvement. The patient was also the subject of extensive urethral disease, on which account, (after he had been for many weeks in attendance at the Ophthalmic Hospital,) I admitted him as an in-door patient, at St. Thomas's Hospital, where, in addition to the alterative medical treatment, I directed the use of the warm bath every other day, for the relief of urethral affection; he was taking at the time, small doses of the bichloride of mercury with sarsaparilla, and had been doing so for several weeks previously, as it appeared to check the progress of the ophthalmic disease, although it had not produced any decided improvement in it. After he had taken the bath two or three times, a very marked alteration, for the better, took place in the eye; and I was much gratified in a few weeks, to find that it was perfectly restored." 243.

Several cases have since done well under treatment of a similar kind. But the preceding may be taken as a sample of the whole.

*Of Inflammation of the Cornea with Deposition of Earthy Matter.*—Mr. Tyrrell believes that the deposit he is about to describe is earthy, and that it has an inflammatory origin.

Most frequently the cornea is partially dotted or speckled with small irregular, dull, and defined opake spots, clustered together, and occupying various positions, in different cases; being sometimes, near the centre, and at others, towards the circumference, but not confined to any part of it. If viewed obliquely, these little patches appear slightly elevated, from the surface of the conjunctiva. If the inflammatory action is still proceeding, the cornea is nebulous, from slight interstitial deposit of fibrin, especially beneath the seat of the superficial disease. This nebula, however, soon subsides, after the inflammation is subdued, when the small opake spots on the surface, become more distinct and defined.

He has occasionally seen this deposit confined to one spot an eighth of an inch in diameter. He has twice removed the substance with a needle, but, unfortunately, did not submit it to chemical analysis. He doubts the correctness of the opinion that these deposits are precipitates from the solutions of metallic salts, particularly those of lead.

" In several instances, I have seen this appearance, when the patient has not previously made use of any metallic preparation. In some, I have seen the deposit increase, when I have been confident that merely tepid water had been applied—and further, I have known a second and third attack of disease, creating this deposit, without any of the solutions alluded to being employed.

Supposing such an appearance to result, from the continued use of a solution of the metallic salts, I consider that it would be a common occurrence at the Ophthalmic Hospital, as the acetate of lead, in solution, is the common lotion prescribed in cases of simple ophthalmia, with ulceration of the cornea; and such cases are very numerous.

The cases, however, in which the cornea exhibits the appearance above described, are extremely rare." 248.

He has seen this disease in children, and in persons of middle age, but never in advanced age—principally in those of scrofulous habit, and, in most instances, also subject to rheumatism or gout.

The first object, in treatment, is the removal of any inflammatory action that exists. He has generally employed a weak solution of the acetic or hydrochloric acid as a lotion, and, with the best effect, in promoting a gradual removal of the deposit. In a few cases, in which the affection of the cornea, has been combined with a chronic ophthalmitis, and the disease has been altogether of long-standing, he has failed, and he has known the eye destroyed by a slow disorganizing process.

*Of Ulcers of the Cornea.*—An ulcer of the cornea, says Mr. Tyrrell, must be in one of the following states.

First, that which we may term healthy, when its surface and circumference exhibit a degree of haziness, or opacity, of a whitish or grey aspect, which is owing to the effusion of adhesive matter on the surface, and in the surrounding texture, which is essential to the healing of the part.

Second, a state in which too much action exists, and which may be recognized by the appearance of small vessels carrying red blood, ramifying into the newly effused matter in the ulcer, which, as described in the first case, renders its surface, and the part immediately around it, nebulous or opake.

The third state, is that in which there is an evident want of action,—when the ulcer appears clear and transparent; merely a small dent presenting itself on a close inspection of the surface, as if a small portion had been cleanly excised by some sharp instrument; there is no apparent deposition of lymph, or any appearance of increased action, as in the two former instances.

The first state, combined with inflammation, indicates that the inflammatory process is not of a very severe kind.

The second shows, that the local action is beyond that which is necessary, and, therefore, that it should be checked.

In the third form, there is a want of action, which shows the surrounding inflammation to be of a chronic or indolent kind, requiring the use of local, as well as general stimulants.

Mr. Tyrrell thinks that on a right understanding of the preceding conditions, hinges the proper mode of treatment. Thus, he says, when the ulcer presents the appearance first described—as the healthy action is proceeding, it is merely required to watch the case, and, by timely application, to prevent any increase of action. In the second instance, the necessity for depletory measures is clearly indicated, and these must be steadily pursued until the visible vascularity of the ulcer is subdued.

In both states, the most simple and inoffensive local application must be employed—as tepid water, or the decoction of poppy-heads, or chamomile flowers. The third form is more difficult to manage. It must first be ascertained whether there is simply a deficiency of local action, or of both local and general. If the former only, the application of mild stimuli only is requisite. When there is feeble constitutional power as well, both local and general remedies are needed. The stimuli employed must be at first weak, only of sufficient strength to create a slight smarting when applied; they are best used in the form of solution, so that they can be thrown immediately on the affected part, by means of a syringe. The application should be repeated, every five or six hours, until a slight haziness around the ulcer, indicates the commencement of the proper action—and, at the same time, the strength of the solution should be increased gradually, or a stronger stimulus employed, each time, until the desired effect is produced. The best stimuli, are the salts of zinc, and the nitrate of silver. Mr. Tyrrell prefers this greatly to the nitrate of silver in substance.

It often happens, says Mr. Tyrrell, that ulceration of the cornea takes place in the chronic stages of purulent or strumous ophthalmia, when a nebulous and vascular condition of the cornea exists, as consequent upon the granular state of the eyelid, or continued scrofulous inflammation.

In such cases the ulcers are generally indolent, being transparent, although many vessels carrying red blood are apparent in the conjunctiva corneæ; so that upon superficial inspection, the presence of these red vessels might be taken as evidence of too much local action, and the ulcers be considered as inflammatory: but if a careful examination be made, the red vessels cannot be traced to the ulcers.

"In addition to the forms of ulcer of the cornea which I have enumerated, two others exist, to which the term sloughing may be applied; the one is dependent on an excess of surrounding action, producing an impediment to the circulation; when a portion of the cornea, losing its vitality, assumes a dense dull opake appearance, and becomes separated, by a process of ulceration, which I have described when speaking of purulent ophthalmia. But little mistake can be made in the treatment of such a case, as the surrounding acute inflammation is generally sufficient, to indicate the proper plan to be pursued.

The other form of sloughing ulcer depends on the want of local action; and the sloughs that are separated from the surface of the ulcer, are of a dirty ash color; they are thrown off in successive thin layers. In this case there is but little, if any, evidence of increased action; and when not covered with slough, the ulcer is clear and transparent." 257.

*Staphyloma Corneæ.*—Mr. Tyrrell in describing the operation for the excision of the staphyloma, observes that "the operation cannot be effected without incising the iris, which is always adherent to the staphylomatous mass; and its vascularity is, in some cases, so much increased, in consequence of the previous disease, that severe hæmorrhage occasionally ensues: to such an extent have I known this occur, that I should be averse to perform the operation on a child of feeble power. The bleeding may be in a measure restrained by cold and pressure; but the patient cannot bear the latter to a sufficient degree, to check the hæmorrhage altogether, and it sometimes continues for many hours.

In performing the operation, a sufficient opening should be made, to allow of the escape of the humors; otherwise, a fresh staphyloma is likely to form; but I should not advise the operator to interfere with the sclerotic tunic, as I have observed that severe suppurative inflammation has followed, in several cases in which that coat has been divided."

"*Partial Staphyloma,*" he adds, "often exists under circumstances, which render its reduction a matter of importance or anxiety, as when there is sufficient of the healthy cornea left to enable the surgeon to form an artificial pupil beneath, or when the projection gives rise to much irritation or deformity. I have succeeded, in several instances, in effecting a reduction of partial staphyloma, by the careful application of nitrate of silver, or hydrate of potash, in substance: I have applied the escharotic first at the base of the projection, taking care not to injure the remaining sound portion of the cornea—the effect has been the separation of a small slough; but previous to such separation, a deposit of fibrin beneath, by which the deeper part has become more solid and strengthened; after the part has recovered, from one application, I have made a second close to, but not upon the same spot, and nearer to the summit of the projection: again and again, I have repeated this operation, acting upon the more prominent part, until a considerable or perfect reduction of the staphyloma has been accomplished; and this has enabled me, in a few cases, to form an artificial pupil, subsequently, of much more utility to the patient. I prefer the hydrate of potash, unless the projection be very small; for its use is followed by a much larger deposit of fibrin, than results from the nitrate of silver. Before applying either, the portion to be used, should be reduced to a fine point; and, when used, the surface should be cleansed from secretion, by a piece of lint; the application should be lightly made; and, immediately afterwards, a little sweet oil should be dropped into the eye, before the eyelids be allowed to close."

*Conical Cornea.*—In the early stage, he thinks it may be retarded, if not prevented from increasing by the local use of stimuli. But, though he knows of nothing that has any beneficial effect upon bad cases, he has succeeded in affording much relief by a very simple plan. This consists in altering the position of the pupil, and removing it from beneath the centre of the cornea, or that part which has its figure most changed, to near the margin, when the least change has occurred; the error in refraction is consequently much lessened, and the vision becomes more perfect, and the focus lengthened.

"I effect the change, in the position of the pupil, in the following manner. I make a puncture, with a broad needle, close to the junction of the cornea and sclerotic; but through the former, and at the part corresponding to the interval between the abductor and depressor muscles; that is, at the outer and lower part of the cornea, (the instrument should be just of sufficient size to effect an aperture merely large enough to admit the passage of a small blunt hook;) I then introduce a small blunt hook, by the aperture in the cornea, and catch the pupillary margin of the iris; and the margin thus caught, I carefully draw out of the aperture by the hook; and, subsequently, as much of the membrane as is requisite to cause the pupillary opening of the iris, to change its position, from the centre to the outer and lower part of the cornea. The portion of the iris, brought out by the hook, I then cut off by a fine pair of scissors, or leave it hanging from the wound, in which part of this membrane is held, and, subsequently, becomes fixed in the cicatrix; whilst the projecting part separates by ulceration or slough. I usually cut off the projecting piece of iris, close to the wound; otherwise, it is apt to create some degree of irritation, by the friction of the eyelid, but should it be left, and irritation arise, it is soon remedied, by touching the portion of iris with nitrate of silver. I have performed this operation seven or eight times; and, in each case, it has benefited the vision, and, in two cases, very considerably. The advantage gained is more than adequate to the risk incurred; for, in no instance, has any evil followed, beyond the slight degree of inflammation, necessary to repair the mischief, occasioned by the operation." 279.

### Sclerotitis.

Passing over Mr. Tyrrell's description and general mode of managing sclerotitis, we may mention the following hints.

In those cases, says Mr. T., in which the constitutional powers are naturally feeble, or have been reduced by medical discipline, a generous diet should be allowed; still, however, without acids, or fermented liquors; the bowels should be kept regular, and mild tonic medicines resorted to. "I have found," he goes on to say, "the most decided and rapid benefit, from the use of small doses of bark and dried carbonate of soda, (five grains of each,) given about every four or six hours. This remedy was mentioned to me, some years ago, by Mr. Wardrop, and it is a very valuable one, inasmuch as I repeatedly find it successful, after the continued, but useless, trial of other means.

It appears necessary to employ the small doses, to produce the beneficial effect: for, in several cases, I have known scruple or half-drachm doses administered without benefit; and the same patients recover quickly, by resorting to the smaller quantities.

One of my colleagues had suffered from a slight attack of sclerotitis, for several weeks, and the disease had baffled the ordinary local and general remedies; tonics had been used freely, and, among them, large doses of bark and soda; the small doses, tried at my suggestion, soon relieved the eye from the diseased action." He has found a change in the tonic serviceable. He prefers warm applications to cold—dry warmth to wet—the former effected by means of small muslin or thin flannel bags, filled loosely with chamomile flowers, and heated in a hot plate or a warming pan.

### Affections of the Aqueous Membrane.

Mr. Tyrrell assures us that three cases have come under his observation, which have proved to him, most satisfactorily, the extension of the aqueous membrane over the iris, continuous with that which lines the posterior surface of the cornea. In each of these cases slight cloudiness of the membrane existed, and the iris appeared dull and incapable of reflecting light, though not altered in color; and small tubercles could be easily perceived, both on the corneal and iritic portions of the membrane, varying from the minutest point to the size of the head of a large pin.

### Inflammation of the Iris.

Mr. Tyrrell denies the justice, and we cannot help thinking that he is right, of those manifold varieties of iritis which ophthalmologists have been so anxious to insist on. " I deem," says he, " such division of the subject to be of no practical utility, with the exception of a division into acute and chronic, which I shall therefore adopt. In fact, I do not admit of the distinctions which have been attempted; but I consider inflammation of the iris to be the same, whatever may be its mode of origin; it may, and does vary in intensity, and in rapidity of progress; and these circumstances are depending more upon the condition of the constitutional power of the party affected, than upon the mode of origin; a specific taint by its influence upon the system, no doubt, in many cases modifies the local disease. I cannot allow, therefore, that idiopathic, traumatic, syphilitic, rheumatic iritis, &c. are distinct diseases, but one and the same affection, generated by different causes."

Mr. Tyrrell is a decided advocate for mercury in acute iritis. " I am so satisfied," he protests, " with the efficacy of mercury, in these cases, that I deem it almost a specific in pure iritis. It is probable, as asserted by some authors, that this affection may be subdued without the aid of mercury, by the ordinary depletory treatment, which is usually employed in common cases of inflammation. I am of opinion, however, that there is considerable risk in such mode of treatment; as I have known many cases, in which it has been pursued to a great extent, and has nevertheless failed in subduing the disease; although it has mitigated its severity, and arrested its progress to a great extent; but a chronic stage has supervened, which has gone on in a slower, but not less certain way, to the ultimate destruction of vision.

The mercurial plan of treatment I consider to be safe and certain, when carefully pursued; the antiphlogistic plan I consider to be uncertain, and therefore unsafe; and if it do succeed, it does not effect a cure in double the time at least, in which mercury annihilates the disease."

Mr. Tyrrell relates two interesting cases of cyst connected with the iris, in the one certainly, in the other supposed to be, dependent on a foreign substance. We quote the first case as the shorter. In both, vision was substantially lost after the performance of an operation.

A singular case of formation of cyst, in connexion with the anterior surface of the iris, occurred in a boy, sent to the Ophthalmic Hospital some time since. The boy had been an apprentice to a blacksmith, and during his work, a small particle of hot iron penetrated his cornea, and lodged in the iris; this gave rise to severe inflammation, which was with difficulty subdued; but he recovered after several weeks, with good vision, but a slightly disfigured pupil. Some months afterwards, a small cyst was formed in connexion with the injured part of the iris, and it continued gradually to increase without suffering or inconvenience, until it acquired the size of a small pea; it was attached near to the pupillary margin of the membrane and projected into the anterior chamber; it was of nearly a round figure, and the surface was shining and white, like a delicate tendinous structure. The boy was sent up to the London Ophthalmic Hospital, under the care of Mr. Scott, who removed the cyst, but useful vision was lost.

*Tremulous Iris* Mr. Tyrrell believes to depend on a partial or complete paralysis of its muscular fibres. This theory he grounds on the fact of there being very little or no motive power in the iris, in all cases which have come under his observation. He affirms that the tremulous motion is always greater when the iris does not expand or contract, so as to change the diameter of the pupil, than when a slight power of contraction and dilatation remains.

*Conjunctivo-Sclerotitis.*—Mr. Tyrrell's account of this is complete. We introduce his remarks on colchicum and turpentine. "*Colchicum,*" says he, "is so uncertain a remedy, that I rarely employ it. I have, however, occasionally known it to afford relief most rapidly; but, I have often used it, without its producing the slightest benefit, as regards the local affection. From the experience I have had respecting it, I should say that it rarely, if ever, does good, when any functional disorder of the stomach or bowels exists; or when the index to these parts, namely, the tongue, is at all loaded or foul; but that when the tongue is clean, and the secretions from the alimentary canal are proper, it will sometimes effect a cure more rapidly than any other remedy I know of. I use it occasionally in the following way. I first act freely upon the bowels by some drastic purge, combined with mercury; and soon after, I direct the patient to take half a drachm of the wine of the colchicum seed, combined with a small quantity of alkali, and some narcotic; and to repeat the dose every six hours. I take care to see the patient after the second or third dose, in order to determine upon the continuance of the remedy; for if it produce nausea, or affect the bowels, it seldom acts beneficially on the ocular disease; but if relief be obtained from the first two or three doses, a cure is usually promoted by perseverance in this treatment. When I prescribe the colchicum, it is usually in the early stage of the disease; not at the period at which I have recommended the use of soda and bark, or quinine; but whilst the conjunctival affection, as well as that of the sclerotic, exists."

Turpentine he has also found so uncertain that he has ceased to prescribe it. He has known it occasionally serviceable in the second stage of the affection, or when the conjunctivitis has been subdued; but he prefers small doses of bark and soda, or of quinine, to it, as more certain and safe remedies; whereas, the turpentine is liable to produce severe and continued distress. He introduces several cases very much in point.

*Sclero-Iritis.*—Mr. Tyrrell gives us his experience, too, on colchicum and turpentine in sclero-iritis. He tells us that he has occasionally employed colchicum in the early stage of the disease with good effect, when the tongue has been clean and the bowels regular; but very seldom are the general secretions in such order, as, in his opinion, to admit of the use of this remedy, with fair chance of success. It is in these cases, also, that turpentine has sometimes proved serviceable; and from which it, as well as bark and soda and other tonics, have obtained credit as remedies for iritis; over which they have little or no influence, but would, in most instances, act injuriously. In sclero-iritis the affection of the iris is usually secondary, consequent on, and in some measure depending upon the sclerotitis, and may sometimes be cured by subduing the sclerotitis or primary disease; but, in most cases, it would be extremely imprudent to trust to the subsidence of the iritis, and treat only the sclerotitis; for though the iritis is first promoted by extension of morbid action from the sclerotic, the inflammatory action is not controlled by the disease of the sclerotic; but once fairly set up it goes on independent, in a great measure, of the sclerotitis. Further, the disease of the iris soon extends to the choroid, and inflammatory action in these textures places the organ in great jeopardy, as regards vision; so much so, that it would be folly not to resort to a remedy, which is almost certain in its effects, in checking the morbid action of the iris and choroid, if it be properly managed, and supposing the disease to be remediable.

### Abscess over the Lachrymal Sac.

Mr. Tyrrell directs attention to a trifling affection which we have seen mistaken.

It is not uncommon, he says, in scrofulous children, to see a small circumscribed abscess immediately over the lachrymal sac, which, from its appearance and the symptoms it gives rise to, might be readily mistaken for disease of the sac itself; for the swelling, either by its pressure on the sac, or by displacing the inferior punctum lachrymale, and interrupting the course of the secretions to the nose, occasions an epiphora; and the notril of the same side appears dry. Independent of diseases in the lachrymal passages being rare in children, the following circumstances will generally explain the true nature of the complaint: the rapidity with which the swelling forms, and its not being preceded by any watering of the eye or epiphora; the early discoloration of the surface of the swelling, and its not having the decided circumscribed and hard feel which abscess of the sac presents; the pain also is very trifling, in comparison with that which accompanies suppurative inflammation in the sac itself. An early opening, but a cautious opening is necessary. And, if abscess of the palpebra point at the upper and outer part of the superior palpebra, the surgeon must be careful not to pass the lancet very deep, as he may wound some of the ducts passing from the lachrymal gland, and occasion a troublesome fistulous sore.

*How to Cure a Black Eye.*—This, as Mr. Tyrrell observes, somewhat grandiloquently, " although an affection of trifling import, it is usually one of considerable importance to the patient unless very young; as it is generally considered a mark of a quarrelsome disposition; and often excites remarks which few can with patience submit to." In plain English, nobody likes to have a black eye. But how to get rid of it? When induced by external force, for the first few hours, cold should be applied; and if the tumefaction become considerable with symptoms of inflammatory action, local bleeding should be also resorted to. As soon as the symptoms of inflammation have subsided, a poultice, composed of the root of the black bryony, finely scraped, after being deprived of its external bark, and mixed with crumb of bread or flour, so as to form it of a proper consistence, should be laid over the discolored part, enclosed in a thin muslin bag; a fresh application should be made every six or eight hours, until the absorption of the effused blood be completed: this will usually take place in forty-eight hours, or a little more, even when the ecchymosis is considerable. "I became acquainted, continues our author, with this remedy, from noticing that some of our celebrated pugilists appeared, a few days after severe encounters, without any disfiguration from ecchymosis; and, on enquiring the reason of this, I found they employed the bryony root, in the manner I have described, to remove such evidence of their occupation. When the bryony root cannot be procured, the absorption may be accelerated by the use of most of the ordinary stimuli, employed in the form of poultice, as oatmeal and vinegar; muriate of ammonia in solution, mixed with bread or linseed; stale beer-grounds, &c."

### Tumors of the Palpebræ.

Mr. Tyrrell first speaks of the small *sebaceous tumor*, which he punctures and squeezes.

Secondly, of the *Glandiform Tumor* which he thus describes. These tumors commence, in a manner very much resembling those last described, excepting that they are less numerous. At first, there is an appearance of a deposition of a white sebaceous matter beneath the cuticle; but the aspect of the tumor soon alters, it increases to a much larger size than that last described; and, occasionally, acquires a magnitude equal to that of a small bean. As it increases, the uniform white surface is interrupted by streaks, and it becomes somewhat mottled. If allowed to proceed, suppuration occurs in its centre, and this occasionally takes place, when it is of small size—not so big as a small pea; or it may not occur, until it has acquired the magnitude mentioned above: when matter forms, it gradually makes its way, by ulceration, through the summit of the swelling; and, after its escape, the cavity formed by the abscess is gradually filled up, by the increase of the morbid growth: and, after the cavity is thus obliterated, the growth is continued through the aperture by which the matter has escaped. This growth is irregular, and resembles very much in appearance the common wart, for which it is very constantly mistaken. These tumors may be readily removed by passing a lancet, or pointed knife, through the tumor, so as to divide it from base to summit, with some part of the surrounding and investing integument. After such division, firm pressure with the nails and extremities of the fore-fingers, on opposite sides of the base of the tumor, will cause it to rise, so that it may be readily detached, by seizing it with a pair of common dissecting forceps; when excised and examined, the morbid growth has a very close resemblance to a portion of a conglomerate gland, as the lachrymal, parotid or pancreas. After the operation, the lead lotion is to be applied.

Thirdly, of *Vesicular Tumors.* They are semi-transparent; and seldom exceed, in size, the volume of a swan shot; occasionally, a single one arises, but more frequently many exist, at the same time, upon one of the lids. A considerable portion of the vesicle should be removed, and some stimulant or escharotic should be applied.

Fourthly, of *Warts of the Palpebræ:* for which he uses a fine ligature, and afterwards, strong acetic acid.

Fifthly, of *Encysted Tarsal Tumors.*

"When small, the tumor is scarcely apparent to external view, and can be only felt, beneath the integument of the palpebra; and the sensation communicated to the finger is such, as if a small shot were seated in the cellular tissue, beneath the skin; it also feels as if loose beneath the integument, and is usually situated near the centre of the tarsus. If, however, the lid be everted, a discolored spot on the inner surface of the tarsus, &c. indicates the point of connexion between the tumor and this structure. The tumor slowly increases; and many weeks or months frequently elapse, from the period at which it may be first felt, until it has acquired sufficient size to be readily perceived, on viewing the outer surface of the palpebra. The integument, immediately above the tumor, is, generally, of the natural color and appearance; unless any acute inflammatory action be set up, then it assumes a redish color. This rarely happens, until the swelling has increased so far as to be readily perceptible. It is at first hard and circumscribed; as it increases, it diminishes in density, and acquires an elastic feel; but is still firm; it seldom exceeds in size the bulk of a common pea. The spot I have alluded to, apparent on the inner surface of the lid, is at first red, and as it increases, the centre exhibits a yellowish cast; and, in the more advanced stage of the disease, a bluish spot occupies the centre; whilst, immediately around this, the redness is still apparent: frequently more than one exists at the same time."

"The diagnosis of this disease is important, as it is most readily cured, when recognised, merely by everting the lid and puncturing the cyst, through the conjunctiva and tarsus; and, immediately that the fluid contents have escaped, breaking up the cyst, with a pointed probe, introduced through the puncture, and forcibly moved about in all directions." 376.

We cannot say that we have seen this plan always answer. We generally introduce a pencil of caustic to destroy the cyst; but even that will occasionally fail.

Sixthly, *of Encysted Tumors of the Palpebræ unconnected with the Tarsus.*—Mr. Tyrrell dissects these carefully out, if possible, without wounding the cyst. But, in those cases in which the cyst is intimately connected with the periosteum—and this is usually indicated by the indentation of the bone, he feels little inclination to meddle with them, as he has seen much mischief the consequence. He introduces several cases, we quote one.

"In one case in which an encysted tumor had existed, in connexion with the frontal bone, near the middle of the superciliary ridge, an attempt was made, I believe, to excise the cyst, but unsuccessfully; the operation was followed by excessive inflammation and sloughing; by which the bone became exposed, and subsequently exfoliated to such an extent that some months after, when the lady was sent to town for my advice, I could touch some extent of the dura mater, from the loss of a large part of the roof of the orbit. I eventually succeeded in closing the wound, and, fortunately, but little deformity resulted." 480.

